# COVID-19 and Psychological Disaster Preparedness – An Unmet Need

**DOI:** 10.1017/dmp.2020.219

**Published:** 2020-06-25

**Authors:** Vishwesh Agarwal, Supriya Sharma, Latika Gupta, Durga Misra, Samira Davalbhakta, Vikas Agarwal, Ashish Goel, Shelley Aggarwal

**Affiliations:** Mahatma Gandhi Mission’s Medical College, Navi Mumbai, India; Sanjay Gandhi Post Graduate Institute of Medical Sciences, Lucknow, India; Byramjee Jeejeebhoy Government Medical College and Sassoon General Hospitals, Pune, India; University College of Medical Sciences, Delhi, India; Stanford University School of Medicine, Stanford, California

**Keywords:** awareness, epidemic, knowledge, psychological preparedness, psychology, risk

## Abstract

**Objective:**

The coronavirus disease (COVID-19) pandemic is a disaster of unprecedented proportions with global repercussions. Psychological preparedness, the primed cognitive awareness and anticipation of dealing with emotional responses in an adverse situation, has assumed a compelling relevance during a health disaster of this magnitude.

**Methods:**

An anonymized eSurvey was conducted in India to assess psychological preparedness toward the ongoing pandemic with a focus on knowledge, management of own and others’ emotional response, and anticipatory coping mechanisms among the survey population. An adapted version of the qualitative Psychological Preparedness for Natural Disaster Scale validated by the World Health Organization was widely circulated over the Internet and various social media platforms for assessment. Results are expressed as median ± standard deviation. Descriptive statistics were used and figures downloaded from surveymonkey.com.

**Results:**

Of the 1120 respondents (M:F 1.7:1, age 35 years ±14.1), most expressed a high level of perceived knowledge and confidence of managing COVID-19, such as awareness of the symptoms of the illness (95.1%), actions needed (94.4%), hospital to report to (88.9%), and emergency contact number (89.1%). A majority (95%) monitored regularly the news bulletins and scientific journals regarding COVID-19. However, nearly one-third (29.2%) could not assess their likelihood of developing COVID-19, and 17.5% were unaware of the difference between a mild and severe infection. Twenty-three percent (23.3%) were unfamiliar with the materials needed in an acute illness situation.

**Conclusion:**

Psychological disaster preparedness is reasonable, although lacking in specific domains. Timely but focused interventions can be a cost-efficient administrative exercise, which federal agencies may prioritize working on.

The coronavirus disease (COVID-19) pandemic is a disaster of unprecedented proportions with global repercussions.^1^ Similar to other disasters, there is great fear regarding personal well-being secondary to community transmission of the disease. Moreover, the inability to confidently project duration and long-term health and economic outcomes poses unique challenges for individuals, communities, and the countries necessitating a very real need for individual, societal, and administrative connection. Psychological preparedness, the primed cognitive awareness and anticipation of dealing with emotional responses in an adverse situation, has assumed a compelling relevance during a health disaster of this magnitude.^2^ Perspectives on mental health in pandemics are sparse and generally limited to the sequelae rather than preparedness to deal with the instigating event.

Densely populated cities, ever increasing global urbanization, expanding slums, and already crumbling health care infrastructure expose unique vulnerabilities in different parts of the country. In an economy grappling with a shortage of workers, low gross domestic product toward the health sector, and limited private sector engagement, providing mental health care takes a backseat over the pressing need to deliver acute medical care. Moreover, the stress of confinement is likely to accentuate mental health problems, including but not limited to anxiety and panic, with limited remedial measures while access to health care is difficult amid a lockdown and availability of pharmaceuticals is poor.^3^ Furthermore, the needs of specific populations, such as the elderly, health care workers, and those with previous mental illnesses, may differ in requirement and priority.^4^ The numerous challenges in treatment make preventive medicine a particularly attractive target. Moreover, a societal construct with strongly interwoven religious, cultural, and ethnic influences necessitates unique and structured approaches to allow percolation of policies through the layers of bureaucracy for timely delivery to the masses. Therefore, care providers and policy-makers face unique challenges with regard to mental health, which continue to evolve through the various stages of the pandemic,^5^ adding to the complexity.

In this context, an anonymized eSurvey was conducted in India to assess psychological preparedness toward the ongoing pandemic with a focus on knowledge, management of emotional response and social environment, and anticipatory coping mechanisms among the survey population.

## METHODS

### Design of the Questionnaire

Overall, the questionnaire featured 29 questions, most (26) of which were multichoice. While 4 items were to identify respondent characteristics, the qualitative Psychological Preparedness for Natural Disaster Scale (PPNDS) by Zulch et al. and validated by the World Health Organization was modified to substitute the word *disaster* with *COVID-19* for assessment of mental preparedness.^6^ The questionnaire is designed to arrive at an indication of how psychologically prepared an individual is for a disaster. It evaluates psychological preparedness in 4 subscales: knowledge (13 questions), management of one’s own emotional response (7 questions), management of others’ responses (3 questions), and awareness and identification of one’s own feelings (3 questions). The 4-factor, 26-item PPNDS scale showed excellent internal consistency, with a Cronbach’s alpha value of α = 0.93. One question could not be modified to suit the pandemic and was deleted (*I am familiar with the disaster warning system messages used for extreme weather events*). Six individuals from the author team participated in the assessment of content validity by scoring each question (as essential, useful, and not required) to obtain a Lawshe’s score of 1.^7^ Hindi translation was carried out by VA, LG, and VA. Back translations were carried out by SS, SD, and DPM. The final Hindi translation was agreed upon by all 6 authors. Following this, the translated version and the original PPNDS were filled by 3 individual respondents to identify errors in wording, grammar or syntax, and critically evaluate the modifications from the original survey.

The average survey time was 5 minutes. The respondents could change the answers before submission but not after it. All questions were mandatory. The survey was completely anonymized.

### Population Selection

The questionnaire was served to the general population across the country. The survey was widely circulated over social media (WhatsApp®, Facebook, Instagram, YouTube, and Twitter with hashtags #COVID #India) to be voluntarily filed by Indians who were 20 years old and above. The approach was all-inclusive for a more wholesome representation of the surveyed population. There was no particular sampling technique used, and all those who agreed to participate were included in the survey. Incomplete responses were excluded. The eligible participants were given a week’s time to voluntarily complete the questionnaire from April 10–17, 2020. Informed consent was taken at the beginning of the survey and no incentives were offered for survey completion.

An exemption from review was obtained from the Institutional Ethics Committee of Sanjay Gandhi Postgraduate Institute of Medical Sciences, Lucknow, as per local guidelines.^8^ We adhered to the Checklist for Reporting Results of Internet E-surveys to report the data. ^9^ Descriptive statistics were used, including figures downloaded from surveymonkey.com^®^. The options “Hardly true for me” and “Not at all true for me” were clubbed together for analysis purposes.

## RESULTS

**FIGURE 1:**
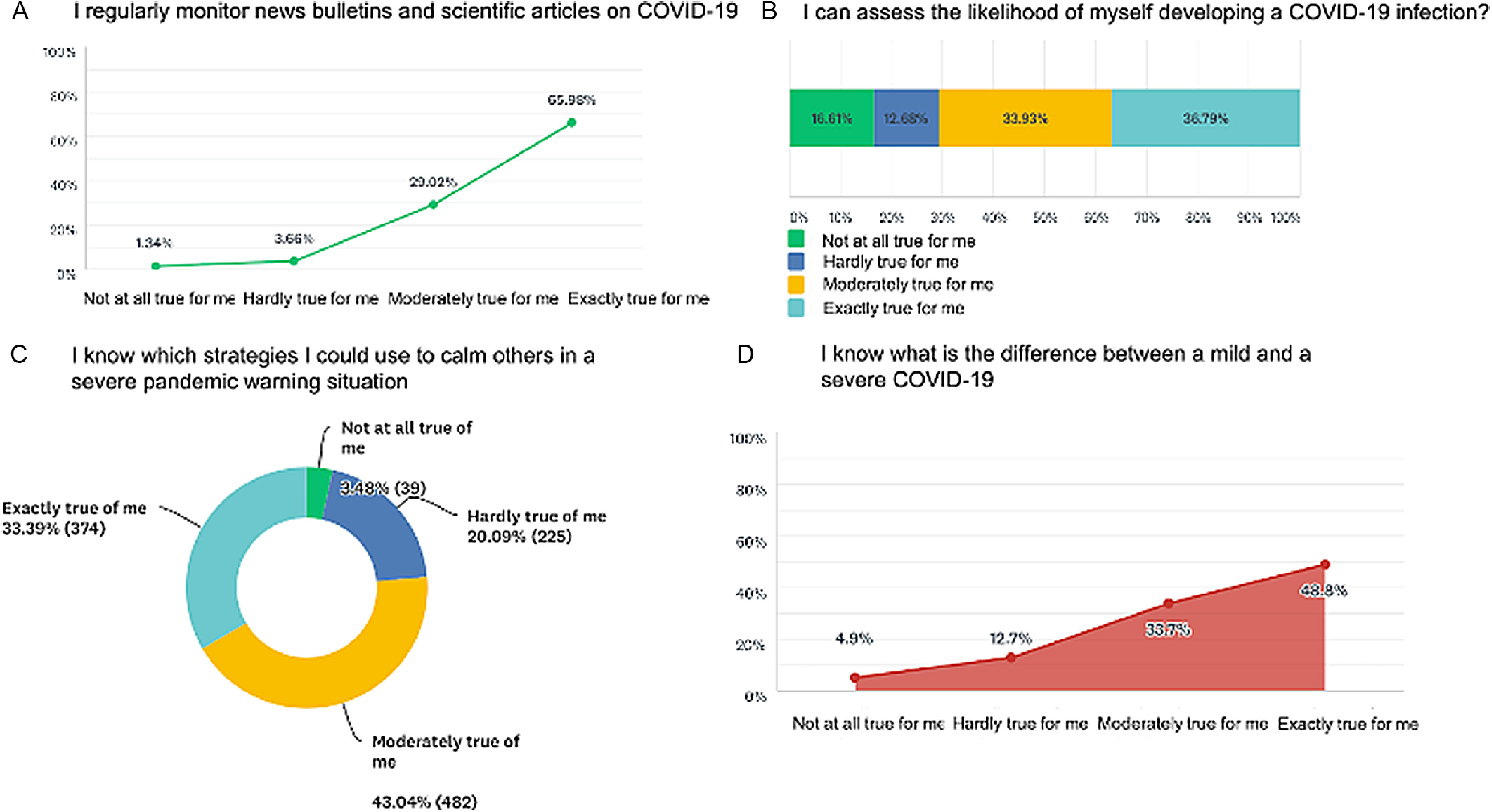
Poor Knowledge and Self-calming Strategies in a Severe Illness Situation.

Of the 1120 respondents (M:F 1.7:1, median age 35 years ±14.1, range 20–87), 710 (63.4%) were living in the hotspots (red zone). Twenty-six (2.3%) were elderly. Thirty-five individuals provided incomplete responses and were, hence, excluded from the analysis. Most expressed a high level of perceived knowledge and confidence of managing COVID-19, such as awareness of the symptoms of the illness (95.1%), actions needed (94.4%), hospital to report to (88.9%), and emergency contact number (89.1%, Supplementary File 1). A majority (95%, Figure 1) monitored regularly the news bulletins and scientific journals regarding COVID-19. However, nearly one-third (29.2%) could not assess their likelihood of developing COVID-19, and 17.5% were unaware of the difference between a mild and severe infection. Twenty-three percent (23.3%) were unfamiliar with the materials needed in an acute illness situation. While 87.5% felt reasonably confident in dealing with stressful situations, 17.9% thought they could not cope with the anxiety of a severe infection, and nearly 1 in 6 (16.3%) were unaware of strategies to calm themselves. While most (86.9%) could quickly identify others in distress, roughly one-fourth (23.5%) were unaware of self-calming strategies. A majority (86.5%) expressed that they are usually prepared for situations that might be difficult or stressful.

Thus, despite most (95%) regularly following recent developments on COVID-19, including scientific literature, a significant knowledge deficit existed in crucial areas such as anticipated risk and differentiating or managing severe illness (see Figure 1). Although anticipatory coping behaviors were intact, many were fraught with fears of distressing situations, and awareness of coping strategies was low.

## DISCUSSION

Chronic adversities in the developing world possibly condition the population’s reflexes to manage challenging situations and increased resilience. The large proportion of our respondent population lived in hotspots, which could have potentially impacted their preparedness. While such people might be more physically vulnerable, they might be psychologically better prepared to handle adversity.^10^ This cognitive ease could be an underutilized resource to further enhance responses toward coping in a disaster situation. Focused interventions, such as identifying specific risks and the features of a severe illness, as well as strategies to manage anxiety in extreme conditions, might improve the mental preparedness of the community in the face of limited resources in India’s socioculturally diverse population.

A recent study on preparedness for earthquakes in Nepal indicated that communities in the developing world demonstrate high resilience, to some extent, through the culture of sharing and helping each other.^11^ Similarly, a secure community support system, also known as “ultrasociality,” is deeply rooted in the social cohesion arising out of unified spiritual and religious beliefs in the Indian subcontinent.^12^ This could be another contributor to better mental preparedness. However, the present circumstances may not be comparable to past events due to the extraordinary fear that has now become commonplace in previously perceived simple, day-to-day interactions with friends and family. Here, in the setting of an infectious disease pandemic that transmits by merely touching objects and talking to another individual, the willingness to engage may translate differently in real life. The reality of biological vulnerability has created universal psychological distress that is poorly understood in reference to COVID-19.

Not everyone is equally affected by the disaster, as communities are complex and so are their vulnerabilities. The physical and emotional challenges faced by health care workers are unique. However, those working in the front line, directly interacting with the most infective patients, would be differently impacted. Certain ethnic groups might respond to the crisis in a different manner and suffer worse outcomes as seen in emerging data from the United States as well as the UK.^13^ We suspect that the impact of mental distress might be higher in children, adolescents, elderly, individuals with mental disorders and lower socioeconomic strata, and groups that have not been evaluated in the current study.^14^ Thus, the complex social equation of mental health warrants more extensive studies targeting all possible (sub)groups within a community with stratified data sets for the better understanding of mental health-related issues.

A strength of our survey is that this is the first step toward understanding psychological disaster preparedness in a low-middle income country with a large sample size. It is subject to biases inherent to a self-report adapted questionnaire pending validation. The respondent population represents a relatively higher socioeconomic status that has a greater online presence. However, online surveys and remote evaluation are presently the only feasible means of maintaining connectivity while distancing socially and mitigating infectious risks. Further, virtual platforms, while providing an essential societal connection, may offer a false sense of security, which could lead to an erroneous self-assessment of the level of mental preparedness toward adversity. Nevertheless, even an apparent reasonable preparedness signifies a state of optimism, which in itself embodies the capacity for successful adaptation with a problem-focused instead of emotion-focused mechanism in the event of severe stress.

Our study is limited by sampling biases, analysis of predictive variables, and lower representation of the elderly and underprivileged. However, this is the first real data from the developing world in the times of COVID-19. It creates a case to explore preparedness in the population on larger scale with a deeper analysis of demographic, social, psychological (anxiety), and economic influence. Our analysis supports that psychological disaster preparedness is reasonable, although lacking in specific domains. A community-based disaster preparedness program could gather volunteers to reinforce ultrasociality through traditional, individual, and spiritual coping strategies. Besides, educating the population about symptoms of severe illness can induce prompt anticipatory responses, which could possibly reduce adverse outcomes. Kar et al., while delineating the conceptual phases of a disaster, include the preceding (warning phase), during and immediately after, and the post-disaster, reiterating the importance of phase-appropriateness of interventions.^15^ A community that understands the warning signs of impending hazards positions itself to better handle the crisis through appropriate mitigation measures and is likely to cope better and resume normal life sooner.

## CONCLUSION

Thus, psychological disaster preparedness is reasonable, although lacking in specific domains, raising the case for focused interventions such as public education to build awareness about the “red flags” for a severe illness and stress-coping techniques in an extreme situation. Federal agencies may prioritize working on such a simple yet cost-efficient administrative exercise with the potential to save millions of lives and vital economic assets. Moreover, mirroring the learning, experience, and success of individual communities in strengthening resilience may find utility in populations around the world and also for future disasters.

## Supplementary material

For supplementary material accompanying this paper visit http://dx.doi.org/10.1017/dmp.2020.219. click here to view supplementary material
