# COVID-19 and Psychological Disaster Preparedness – An Unmet Need – Corrigendum

**DOI:** 10.1017/dmp.2020.365

**Published:** 2020-06

**Authors:** Vishwesh Agarwal, Supriya Sharma, Latika Gupta, Durga Prasanna Misra, Samira Davalbhakta, Vikas Agarwal, Ashish Goel, Shelley Aggarwal

**Keywords:** awareness, epidemic, knowledge, psychological preparedness, psychology, risk, corrigendum

The name of author Durga Prasanna Misra was incorrect in the original publication of this article.^1^ The article has since been corrected.
