# Supplementary material for: COVID-19 and Psychological Disaster Preparedness – An Unmet Need
Source: Disaster Med Public Health Prep. 2020 Jun 25:1–4. doi: 10.1017/dmp.2020.219 (PMC7385318; doi:10.1017/dmp.2020.219)
Supplement: Supplementary file 1 [file S1935789320002190sup001.docx]

| **Knowledge and Management of the External Situational Environment** | |
| --- | --- |
| **Question** | **Response** |
| **I can assess the likelihood of myself developing a COVID-19**  **Infection?** |  |
| Not at all true of me | 186 (16.6%) |
| Hardly true of me | 142 (12.6%) |
| Moderately true of me | 380 (33.9%) |
| Exactly true of me | 412 (36.7%) |
| **I regularly monitor news bulletins and scientific articles on COVID-19** |  |
| Not at all true of me | 15 (1.3%) |
| Hardly true of me | 41 (3.6%) |
| Moderately true of me | 325 (29%) |
| Exactly true of me | 739 (65.98%) |
| **I know what to do and what actions to take when I develop the**  **infection** |  |
| Not at all true of me | 17 (1.5%) |
| Hardly true of me | 45 (4%) |
| Moderately true of me | 295 (26.3%) |
| Exactly true of me | 763 (68.1%) |
| **I would be able to locate the right hospital to visit when I develop the**  **infection** |  |
| Not at all true of me | 30 (2.6%) |
| Hardly true of me | 94 (8.3%) |
| Moderately true of me | 242 (21.6%) |
| Exactly true of me | 754 (67.3%) |
| **I know how to adequately prepare my home for the forthcoming illness** |  |
| Not at all true of me | 21 (1.8%) |
| Hardly true of me | 113 (10.1%) |
| Moderately true of me | 423 (37.7%) |
| Exactly true of me | 563 (50.2%) |
| **I know where I can quickly find the emergency contact information in a**  **severe illness situation** |  |
| Not at all true of me | 21 (1.8%) |
| Hardly true of me | 101 (9%) |
| Moderately true of me | 299 (26.7%) |
| Exactly true of me | 699 (62.4%) |
| **I am familiar with the materials needed in severe infection that are**  **available to me** |  |
| Not at all true of me | 69 (6.1%) |
| Hardly true of me | 192 (17.1%) |
| Moderately true of me | 409 (36.5%) |
| Exactly true of me | 450 (40.1%) |
| **I know which household preparedness measures are needed to stay**  **safe if a member in the house tests positive** |  |
| Not at all true of me | 36 (3.2%) |
| Hardly true of me | 148 (13.2%) |
| Moderately true of me | 400 (35.7%) |
| Exactly true of me | 536 (47.8%) |
| **I am familiar with the signs and symptoms of a severe COVID-19**  **illness** |  |
| Not at all true of me | 9 (0.8%) |
| Hardly true of me | 45 (4%) |
| Moderately true of me | 252 (22.5%) |
| Exactly true of me | 814 (72.6%) |
| **I know what to look out for (e.g. money, packing, care of children, etc.**  **in my home and workplace) if an emergency illness situation should**  **develop** |  |
| Not at all true of me | 23 (2%) |
| Hardly true of me | 134 (11.9%) |
| Moderately true of me | 442 (39.4%) |
| Exactly true of me | 521 (46.5%) |
| **I know what is the difference between a mild and a severe COVID-19**  **infection** |  |
| Not at all true of me | 55 (4.9%) |
| Hardly true of me | 142 (12.6%) |
| Moderately true of me | 377 (33.6%) |
| Exactly true of me | 546 (48.7%) |
| **I am knowledgeable about the impact that the infection can have on**  **my home** |  |
| Not at all true of me | 19 (1.7%) |
| Hardly true of me | 57 (5.1%) |
| Moderately true of me | 271 (24.2%) |
| Exactly true of me | 773 (69%) |
| **I feel reasonably confident in my own ability to deal with stressful**  **situations that I might find myself in** |  |
| Not at all true of me | 34 (3%) |
| Hardly true of me | 106 (9.4%) |
| Moderately true of me | 438 (39.1%) |
| Exactly true of me | 542 (48.4%) |
| **Management of one’s Emotional and Psychological Response** | |
| **Question** | **Response** |
| **I feel reasonably confident in my own ability to deal with stressful**  **situations that I might find myself in** |  |
| Not at all true of me | 34 (3%) |
| Hardly true of me | 106 (9.4%) |
| Moderately true of me | 438 (39.1%) |
| Exactly true of me | 542 (48.4%) |
| **In a severe infection situation I would be able to cope with my anxiety**  **and fear** |  |
| Not at all true of me | 53 (4.7%) |
| Hardly true of me | 148 (13.2%) |
| Moderately true of me | 415 (37%) |
| Exactly true of me | 504 (45%) |
| **I think I am able to manage my feelings pretty well in difficult and**  **challenging situations** |  |
| Not at all true of me | 30 (2.6%) |
| Hardly true of me | 143 (12.7%) |
| Moderately true of me | 439 (39.2%) |
| Exactly true of me | 508 (45.3%) |
| **When necessary, I can talk myself through challenging situations** |  |
| Not at all true of me | 25 (2.2%) |
| Hardly true of me | 100 (8.9%) |
| Moderately true of me | 412 (36.7%) |
| Exactly true of me | 583 (52.1%) |
| **I seem to be able to stay cool and calm in most difficult situations** |  |
| Not at all true of me | 35 (3.1%) |
| Hardly true of me | 146 (13%) |
| Moderately true of me | 444 (39.6%) |
| Exactly true of me | 495 (44.2%) |
| **I know which strategies I could use to calm myself in a severe illness**  **situation** |  |
| Not at all true of me | 36 (3.2%) |
| Hardly true of me | 147 (13.1%) |
| Moderately true of me | 456 (40.7%) |
| Exactly true of me | 481 (42.9%) |
| **If I found myself at a COVID-19 hotspot I would know how to manage**  **my own response to the situation** |  |
| Not at all true of me | 23 (2%) |
| Hardly true of me | 110 (9.8%) |
| Moderately true of me | 423 (37.7%) |
| Exactly true of me | 564 (50.3%) |
| **Management of one’s Social Environment** | |
| **Question** | **Response** |
| **I would be able to tell easily if others around me are in distress** |  |
| Not at all true of me | 19 (1.7%) |
| Hardly true of me | 127 (11.3%) |
| Moderately true of me | 448 (40%) |
| Exactly true of me | 526 (46.9%) |
| **If others are in distress, I would know how to calm them down** |  |
| Not at all true of me | 24 (2.1%) |
| Hardly true of me | 182 (16.2%) |
| Moderately true of me | 469 (41.8%) |
| Exactly true of me | 445 (39.7%) |
| **I know which strategies I could use to calm others in a severe**  **pandemic warning situation** |  |
| Not at all true of me | 39 (3.4%) |
| Hardly true of me | 225 (20.1%) |
| Moderately true of me | 482 (43%) |
| Exactly true of me | 374 (33.4%) |
| **Anticipatory Coping with Emotional Response** | |
| **Question** | **Response** |
| **I am able to identify my feelings pretty well in challenging situations** |  |
| Not at all true of me | 17 (1.5%) |
| Hardly true of me | 110 (9.8%) |
| Moderately true of me | 444 (39.6%) |
| Exactly true of me | 549 (49%) |
| **During severe illnesses I would notice if I am feeling anxious or**  **stressed** |  |
| Not at all true of me | 21 (1.8%) |
| Hardly true of me | 89 (7.9%) |
| Moderately true of me | 454 (40.5%) |
| Exactly true of me | 556 (49.6%) |
| **I usually prepare mentally for situations that might be difficult or**  **stressful** |  |
| Not at all true of me | 25 (2.2%) |
| Hardly true of me | 126 (11.2%) |
| Moderately true of me | 442 (39.4%) |
| Exactly true of me | 527 (47.1%) |
